# Multiple recognition of RXLR effectors is associated with nonhost resistance of pepper against *Phytophthora infestans*

**DOI:** 10.1111/nph.12861

**Published:** 2014-06-02

**Authors:** Hyun-Ah Lee, Shin-Young Kim, Sang-Keun Oh, Seon-In Yeom, Saet-Byul Kim, Myung-Shin Kim, Sophien Kamoun, Doil Choi

**Affiliations:** 1 Department of Plant Science, College of Agriculture and Life Sciences, Seoul National UniversitySeoul, 151-921, Korea; 2 Plant Genomics and Breeding Institute, Seoul National UniversitySeoul, 151-921, Korea; 3 The Sainsbury Laboratory, Norwich Research ParkNorwich, NR4 7UH, UK

**Keywords:** multiple interactions, nonhost resistance, pepper, *Phytophthora infestans*, RXLR effectors

## Abstract

- Nonhost resistance (NHR) is a plant immune response to resist most pathogens. The molecular basis of NHR is poorly understood, but recognition of pathogen effectors by immune receptors, a response known as effector-triggered immunity, has been proposed as a component of NHR.
- We performed transient expression of 54 *Phytophthora infestans*RXLR effectors in pepper (*Capsicum annuum*) accessions. We used optimized heterologous expression methods and analyzed the inheritance of effector-induced cell death in an F_2_ population derived from a cross between two pepper accessions.
- Pepper showed a localized cell death response upon inoculation with *P. infestans*, suggesting that recognition of effectors may contribute to NHR in this system. Pepper accessions recognized as many as 36 effectors. Among the effectors, PexRD8 and Avrblb2 induced cell death in a broad range of pepper accessions. Segregation of effector-induced cell death in an F_2_ population derived from a cross between two pepper accessions fit 15 : 1, 9 : 7 or 3 : 1 ratios, depending on the effector.
- Our genetic data suggest that a single or two independent/complementary dominant genes are involved in the recognition of RXLR effectors. Multiple loci recognizing a series of effectors may underpin NHR of pepper to *P. infestans* and confer resistance durability.

## Introduction

Plants are challenged by numerous pathogens including fungi, bacteria and viruses. However, most plants are resistant to most pathogens and disease is the exception in nature (Huitema *et al*., 2003). This phenomenon is known as nonhost resistance (NHR), an immune response of plant species against all isolates of a microorganism that cause disease on other plant species (Sumit *et al*., 2012). NHR is the most durable and strong resistance form of plants (Heath, 2000; Gurr & Rushton, 2005), and may have the potential to improve crop resistance to pathogens (Fan & Doerner, 2012). However, the molecular mechanism underlying NHR remains to be elucidated, because classical genetic approaches are limited as most nonhost plants cannot be crossed with susceptible host plants (Niks & Marcel, 2009).

The mechanism of NHR comprises of several components, including pre-formed and induced immunity (Thordal-Christensen, 2003). Pre-formed immunity includes physical barriers, such as cuticular wax, and chemical, antimicrobial compounds. Induced immunity includes pathogen-associated molecular pattern (PAMP)-triggered immunity (PTI) and effector-triggered immunity (ETI) (Thordal-Christensen, 2003; Zhao *et al*., 2005; Jones & Dangl, 2006). PAMPs are conserved components of pathogens that are recognized by pattern recognition receptors at the surface of plant cells, which activate basal defense responses referred collectively as PTI. To suppress PTI, pathogens secrete effectors into plant cells that are specifically recognized by plant resistance (R) proteins leading to ETI (Jones & Dangl, 2006). Defense responses induced by PTI and ETI involve an oxidative burst and transcriptional reprogramming, mediated by signaling machinery (Tsuda & Katagiri, 2010). However, ETI can be distinguished from PTI by the induction of localized programmed cell death in the former, known as the hypersensitive response (HR) (Hammond-Kosack & Jones, 1997; Heath, 2000; Cunnac *et al*., 2009). The relative contribution of ETI and PTI to NHR may relate to the evolutionary divergence time between host and nonhost plants. It was suggested that the relative contribution of ETI increases with decreasing phylogenetic divergence time between two plant species (Schulze-Lefert & Panstruga, 2011).

There is some evidence to show the involvement of ETI in NHR. *HopQ1-1*, a type III effector of *Pseudomonas syringae* pv. tomato, elicited HR in nonhost *Nicotiana benthamiana* and a *HopQ1-1* deletion strain caused disease in the nonhost plants, suggesting a role for ETI in NHR (Wei *et al*., 2007). Similarly, *HopAS1* broadly present in *P. syringae* strains triggered NHR in *Arabidopsis*, whereas pathogenic strains had a truncated form of *HopAS1* (Sohn *et al*., 2012). Resistance genes may also play a role in NHR. *Rxo1* identified from maize encodes R protein that interacts with *avrRxo1* and confers resistance against the nonhost pathogen *Xanthomonas oryzae* pv. *oryzicola* which causes bacterial leaf streak in rice (Zhao *et al*., 2005). When *Rxo1* was transferred to susceptible rice, multiplication of the bacteria was inhibited. Another R protein in NHR is WRR4, which was identified from *Arabidopsis* ecotype Col-0 infected with *Albugo candida* using a natural variation in immunity of *Arabidopsis* accessions (Borhan *et al*., 2008). Transfer of *WRR4* to susceptible *Brassica napus* rendered the plant resistant to *A. candida* (Borhan *et al*., 2010). However, nonhost plants carrying a nonfunctional copy of *WRR4* retained resistance to the pathogen, suggesting polygenic control of NHR (Borhan *et al*., 2008).

*Phytophthora infestans*, an oomycete pathogen, is one of the most devastating pathogens in the world. It causes potato late blight on Solanaceae crops such as potato and tomato, but not on pepper. Oomycete pathogens secrete effectors into host cells that suppress host defense responses and modify host cell processes (Dodds & Rathjen, 2010; Bozkurt *et al*., 2012). The effectors have conserved RXLR motifs in their N-terminal domains that play an important role in targeting the host cells, and they contain W, Y and L motifs in their C-terminal domains (Whisson *et al*., 2007; Jiang *et al*., 2008). The genomic sequence of *P. infestans* reveals *c*. 550 genes encoding possible RXLR effectors. Most of these are found in the expanded repetitive DNA-rich regions, suggesting that they have rapidly evolved (Haas *et al*., 2009; Win *et al*., 2012). Using genomic information, a collection of nonredundant RXLR effectors of *P. infestans* has been cloned into *Potato virus X* (PVX)-based transient expression vectors (Torto *et al*., 2003; Vleeshouwers *et al*., 2008; Oh *et al*., 2009). High-throughput screening systems, such as PVX agro-infection, have been optimized in Solanaceae plants, allowing rapid identification of cognate R genes that interact with RXLR effectors (Takken *et al*., 2000; Torto *et al*., 2003; Vleeshouwers *et al*., 2006). Two of these genes are *Rpi-blb1*, which interacts with *Avrblb1* (Vleeshouwers *et al*., 2008), and *Rpi-blb2*, which recognizes *Avrblb2* (Oh *et al*., 2009). This genomic information and the development of experimental methods that enable effector-omics analyses of plant–microbe interactions, provide a promising approach for studying nonhost interaction.

Here, we hypothesized that multiple interactions between nonhost plant factors and pathogen effectors establish durable NHR. To test this hypothesis, we optimized *in planta* transient expression methods to screen interactions between pepper accessions and RXLR effectors of *P. infestans*. Using the results from the screening, we performed genetic analyses on an F_2_ population to investigate how plant factor(s) control effector-induced cell death. From the inheritance study, we conclude that multiple interaction between plant factor(s) and effectors of *P. infestans*, could be one of the determinants of the durable nature of NHR in pepper against *P. infestans*.

## Materials and Methods

### Plant materials and growth conditions

In order to screen nonhost interactions between pepper and RXLR effectors of *P. infestans*, a total of 100 pepper accessions from various regions were kindly provided by Dr Byoung-Cheorl Kang (Seoul National University, Korea). Seeds of the pepper accessions were sterilized with 0.1% sodium hypochlorite (NaOCl). Seven days after germination at 30°C, all of the seedlings were transferred to controlled chambers with a 16 h : 8 h, light : dark period at room temperature until cotyledons were fully expanded. Four plants from each accession were transplanted into a 32-plug form tray filled with horticultural bed soil composed of cocopeat (65–70%), peat moss (8–12%), and vermiculite (10–14%) according to the manufacturer's information (Baroker, Seoul Bio Co., Ltd, Seoul, Korea).

### *Phytophthora infestans* spore infection

For *P. infestans* inoculation assays, 4-wk-old *Capsicum annuum* L. cv CM334 (nonhost), *Solanum lycopersicum* L. cv Heinz (susceptible host) and *Solanum tuberosum* L. cv Daeji (susceptible host) were used. The *P. infestans* isolates 88069, 40707, 40718, 43071 and 43072 provided by the RDA Gene Bank of Korea were used. All isolates were grown on rye sucrose agar media as previously described at 17°C in the dark for 10 d (Kamoun *et al*., 1998). To release the zoospores from sporangia, the plates were flooded with autoclaved distilled cold water, gently rubbed with a sterile cell scraper, and incubated at 4°C for 1.5 h. The zoospores were counted under a hemocytometer, and the concentration was adjusted to 5 × 10^4^ zoospores ml^−1^. The detached leaves of pepper, potato and tomato were placed in the rectangle plate overlaid with a wet paper towel to maintain high humidity. The 10-μl droplets of zoospores were applied onto the detached leaves and incubated at 17°C. The inoculated leaves were sampled at 0, 24, 48 and 72 h post-inoculation (hpi).

### Trypan blue staining

Upon inoculation of *P. infestans*, leaf discs containing zoospore droplets were excised at 5 d post-inoculation (dpi) and stained with lactophenol-trypan blue solution (10 ml lactic acid, 10 ml glycerol, 9.3 ml phenol, 10 ml distilled water, and 20 mg trypan Blue) diluted 1 : 2 in ethanol under vacuum as described (Yeom *et al*., 2012). The leaf discs were boiled in the lactophenol-trypan solution, incubated overnight in staining solution, and cleared with chloral hydrate (2.5 g ml^−1^). The cleared leaf discs were mounted in 50% glycerol solution and observed using stereo microscopy (Dimis-M, Siwon Optical Technology Co., Ltd, Anyang, Korea) and differential interference contrast microscopy.

### Reverse transcription-PCR

Six leaf disks were harvested using a cork borer from the detached leaves inoculated with *P. infestans*. Total RNA were extracted using the Trizol reagent (Invitrogen) and 2 μg of total RNA were reverse-transcribed using Superscript II (Invitrogen). To determine *in planta* effector expression, PCR was performed using gene-specific primers (10 pmol μl^−1^, listed in Supporting Information Table S1). The following cycling conditions were used: 1 cycle of 94°C for 3 min; 30 cycles (or 35 cycle for pepper) of 94°C for 20 s, 58°C for 20 s and 72°C for 45 s. The actin gene (designated *ActA*) for the pathogen was used as control for monitoring transcript levels (Bos *et al*., 2010). The PCR products were then analyzed on 2% agarose gel and visualized by ethidium bromide.

### PexRD effectors of *P. infestans*

All effectors were cloned into a binary *Potato Virus X*-based pGR106 vector in *A. tumefaciens* strain GV3101 (Holsters *et al*., 1980; Jones *et al*., 1999) for *in planta Agrobacterium*-mediated transient expression. Both pGR106-dGFP and pGR106-PexRD2 were used as negative and positive controls, respectively.

### PVX agro-infection

Recombinant *Agrobacterium* carrying pGR106-PexRD effectors was incubated at 28°C on a YEP agar plate containing kanamycin (50 mg l^−1^) and rifampicin (50 mg l^−1^) for 2 d. Using toothpicks, the *Agrobacterium* was inoculated by piercing 5-wk-old pepper leaves. Duplicate experiments were performed twice on different days. Cell death induced by effectors was monitored at 14 dpi.

### *In planta* expression using recombinant PVX virions

*Agrobacterium* carrying PexRD effector genes in binary vector pGR106 were cultured overnight at 28°C in YEP liquid media with antibiotics as described above and resuspended in infiltration buffer (10 mM MgCl_2_, 10 mM MES and 150 μM acetosyringone). The culture was diluted to a final OD_600_ of 0.5 and incubated with gentle shaking at room temperature for 3 h, then infiltrated into the expanded leaves of *N. benthamiana* with a 1-ml disposable syringe without a needle. At 7 dpi, a 1-g sample of upper leaves that emerged after systemic infection of PVX was collected and ground in 5 ml of 0.05 M potassium phosphate buffer (pH 7.4) using a pestle and mortar to make the sap inoculum carrying recombinant PVX virions. The inoculum was rubbed onto the leaves of 4-wk-old pepper plants using carborundum 400 mesh. Each virion was applied to two leaves of each plant to prevent virions from being mixed. Then, distilled water was applied onto the inoculated leaves to wash out the carborundum. Cell death on the inoculated leaves was monitored up to 7 dpi, and necrotic cell death was observed by destaining chlorophyll with 100% ethanol.

## Results

### Nonhost resistance of pepper against *P. infestans* is associated with hypersensitive cell death

**Fig. 1:**
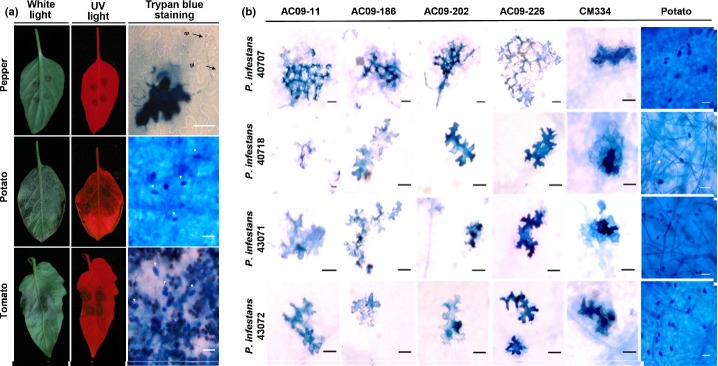
Nonhost response of pepper (*Capsicum annuum*) against *Phytophthora infestans* is associated with hypersensitive cell death. (a) Detached leaves of nonhost pepper landrace CM334 and host plants (potato (*Solanum tuberosum*) cv Daeji and tomato (*S. lycopersicum*) cv Heinz) were infected with a zoospore suspension of *P. infestans* 88069 (5 × 10^4^ ml^−1^). Leaf pieces containing zoospore droplet were excised at 5 d post-inoculation, examined for hypersensitive cell death and sporangia development under UV illumination, and visualized by trypan blue staining. White arrows indicate sporangia formation on potato and tomato leaves. Zp, zoospore; gt, germ tube. (b) Hypersensitive cell death was confirmed by interactions between pepper accessions and four isolates of *P. infestans* 40707, 40718, 43071 and 43072. Bars, 50 μm.

In order to investigate how nonhost pepper plants respond to *P. infestans* and characterize its nonhost defense response, the detached leaves of pepper, tomato and potato plants were inoculated with a zoospore suspension of *P. infestans* isolate 88069 (5 × 10^4^ spores ml^−1^). The interaction with *P. infestans* was examined cytologically using UV light and trypan blue staining to visualize dead cells. At 5 dpi, dead cells at the infection site were readily shown by their brown color under white light and auto-fluoresced on a red background under UV light. The germinated zoospores of *P. infestans* were shown and germ tubes were elongated on pepper leaves (Fig. 1a, upper right). The HR on penetrated epidermal cells was apparent on pepper leaves and remained limited to those cells (Fig. 1a, upper right). In contrast to the responses in pepper, the biotrophic growth of *P. infestans* was apparent 5 dpi in tomato and potato leaves and hyphae with sporangia, necrotic death of mesophyll cells spread throughout the leaves (Fig. 1a, lower right panel).

NHR is defined as resistance displayed by all accessions of a plant species against all strains of a pathogen. To further examine the pepper–*P. infestans* interaction, we tested five accessions of pepper with four isolates of *P. infestans* (40707, 40718, 43071 and 43072) collected from different regions of Korea (Fig. 1b). All accessions displayed localized cell death typical of the HR to all four isolates. The HR was limited to penetrated epidermal cells in all accessions inoculated with three of the isolates, whereas in pepper infected with isolate 40707, the induced HR was not completely confined to the infected cells and extended to adjacent cells. The finding that NHR of pepper to *P. infestans* is associated with the HR suggests the possible presence of pepper R genes that interact with *P. infestans* effectors.

### Nonhost pepper shares expression profiles of the effector genes of *P. infestans* with host plants

**Fig. 2:**
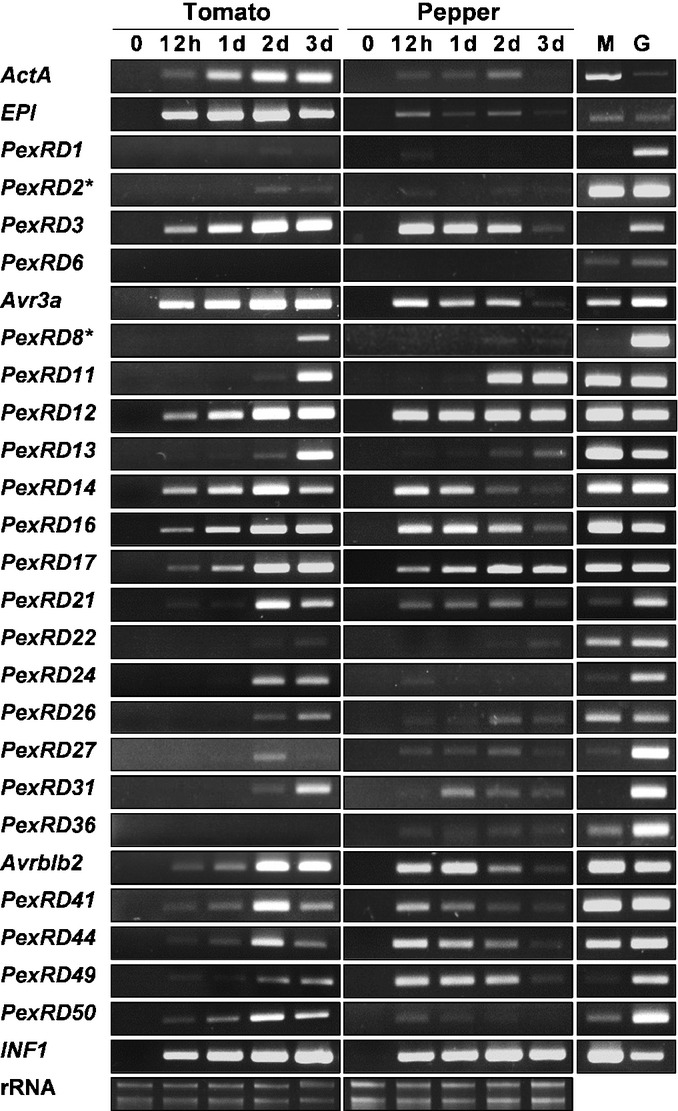
Nonhost pepper shares expression profiles of the effector genes of *Phytophthora infestans* with host plants. Detached leaves of pepper (*Capsicum annuum*) and tomato (*Solanum lycopersicum*) upon inoculation with *P. infestans* isolate 43072 were harvested at 0, 12 h, and 1, 2 and 3 days post-inoculation. Total RNA was extracted from the harvested samples. Reverse transcription-PCR was performed to observe *in planta* expression of PexRD effector genes. Tomato cDNAs was subjected to a 30-cycle PCR and a 35-cycle PCR was performed with pepper cDNAs. Constitutive *ActA* and *in planta*-induced *EPI1* were used as controls and those genes were detected using 30-cycle PCR in both pepper and tomato. This experiment was repeated twice with similar results. M, *P. infestans* mycelium RNA; G, *P. infestans* genomic DNA. *PexRD2 and PexRD8 in tomato cDNAs: subjected to a 35-cycle PCR.

We first examined whether effector genes of *P. infestans* are expressed during the nonhost interaction. The 54 RXLR effectors used in this study can be grouped into 32 PexRD genes of homologs in Table 1. Expression of 24 of 32 PexRD genes was investigated in pepper (AC09-226) and tomato (cv Heinz) leaves following infection with *P. infestans* 43072 using gene-specific primers (Fig. 2; Table S1)*. ActA* expression increased during the infection in tomato but decreased in pepper at 3 dpi, reflecting the susceptible and resistant responses of each plant species. The Kazal-like serine protease inhibitor *EPI1* is an *in planta*-induced gene that may be involved in suppression of PTI (Tian *et al*., 2005; Attard *et al*., 2008). *EPI1* was expressed in *P. infestans*-infected pepper.

**Table 1:** Description of the tested PexRD genes*

Effector names	PITG gene ID	Number of homologs tested	RXLR	dEER	Average number of accessions with cell death
*PexRD1*	PITG_15287	1	RQLR	EDGEER	0
*PexRD2*	PITG_21422, PITG_11350, PITG_11383, PITG_11384, PITG_22935	1	RLLR	ENDDDSEAR	43
*PexRD3*	PITG_09160	1	RFLR	EGDNEER	13
*PexRD4*	NA	1	RFLR	DEER	2
*PexRD6, ipiO, Avrblb1*	PITG_21388	3	RSLR	DEER	5
*PexRD7, Avr3a*	PITG_14371	2	RLLR	EENEETSEER	7
*Pex147-2, Avr3a paralog*		1	RLLR	EESEETSEER	6
*Pex147-3, Avr3a paralog*		1	RFLR	EENEETSEER	3
*PexRD8*	PITG_14739	1	RLLR	DDDDEEER	22
*PexRD10*	PITG_23206	1	RKLR	EER	4
*PexRD11*	PITG_23206	2	RLLR	DEGELTEER	3
*PexRD12*	PITG_16233, PITG_16240	2	RSLR	DSDDGEER	7
*PexRD13*	PITG_08812	2	RQLR		7
*PexRD14*	PITG_14961, PITG_14962, PITG_14954, PITG_14959	2	RLLR	ETGNQEER	6
*PexRD16*	PITG_06087	2	RSLR	EER	4
*PexRD17*	PITG_08599	2	RVLR	EIEAETER	1
*PexRD21*	PITG_13452	1	RLLR	EREVQEER	2
*PexRD22*	PITG_13306	2	RFLR	EDASDEER	4
*PexRD24*	PITG_04314	2	RSLR	ETSEDEEER	8
*PexRD26*	PITG_07947	2	RVLR	DEER	1
*PexRD27*	PITG_13628	1	RLLR	DSEER	0
*PexRD28*	PITG_03192	1	RSLR	ETSEDEEER	1
*PexRD31*	PITG_16402	1	RSLR	EDQEGDEER	2
*PexRD36*	PITG_23132	2	RHLR	DDEER	3
*PexRD39/40, Avrblb2*	PITG_20300	3	RSLR		19
*PexRD41*	PITG_04089	3	RSLR		3
*PexRD44*	PITG_17063	1	RFLR	QEEGVFEER	2
*PexRD45*	PITG_09632	2	RSLR		2
*PexRD46*	PITG_18685	3	RSLR		3
*PexRD49*	PITG_05750	1	RLLR	EEER	3
*PexRD50*	PITG_06099	2	RLLR		2
*PexRD51*		2	RFLR	EER	0

* Table modified from Oh *et al*. (2009).

Of 24 PexRD genes, the expression of 23 PexRD genes was detected in the interaction between pepper and *P. infestans*. Even considering the different cycles of PCR amplification, all of the PexRD genes expressed in tomato were also detected in the pepper–*P. infestans* interaction. Twelve effectors of *PexRD3*, *Avr3a*, *PexRD12*, *PexRD14*, *PexRD16*, *PexRD17*, *PexRD21*, *Avrblb2*, *PexRD41*, *PexRD44*, *PexRD49* and *PexRD50* were expressed in pepper at 12 hpi. The expression of those effectors increased in tomato from 12 hpi. *PexRD8*, *PexRD11*, *PexRD13* and *PexRD22* showed detectable expression in pepper after 2 dpi, which is similar to the expression in tomato. In conclusion, the expression of all the tested *P. infestans* effector genes during interactions with pepper indicates that these effectors could be targeted by the pepper immune response.

### Optimization of an heterologous effector expression system in pepper

**Fig. 3:**
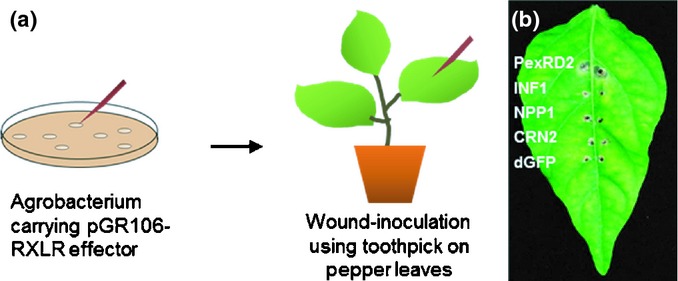
Optimization of an heterologous effector expression system in pepper (*Capsicum annuum*). (a) The PVX agro-infection method is depicted. *Agrobacterium* carrying pGR106-RXLR effectors was wound-inoculated onto pepper leaves. (b) PexRD2 and elicitins (INF1, NPP1 and CRN2) which are known to induce cell death were inoculated on both sides of mid veins in pepper leaves. PexRD2 but not elicitins induced cell death on inoculated pepper leaves at 14 d post-inoculation .

Effector-omics or high-throughput approaches for the rapid assignment of activities to effector genes require effective heterologous expression systems. To establish such a system in pepper, we applied a PVX agro-infection method for the *in planta* expression of effectors in pepper, as depicted in Fig. 3(a). This method is well suited for high-throughput screening of effector activity in Solanaceae plants. To confirm the suitability of this approach in pepper, four elicitors known to induce the necrotic response in Solanaceae plants, specifically INF1 (Kamoun *et al*., 1998), PiNPP1 (Kanneganti *et al*., 2006), CRN2 (Torto *et al*., 2003) and PexRD2 (Oh *et al*., 2009), were wound-inoculated on leaves of 4-wk-old pepper plants. *Agrobacterium* strain GV3101 carrying those elicitors and negative control dGFP were inoculated onto both sides of the mid-vein of fully expanded pepper leaves using toothpicks. Among the four different elicitors, only PexRD2 caused cell death on pepper leaves at 14 dpi (Fig. 3b). Considering the induced cell death by PexRD2, the PVX agro-infection method is as applicable in pepper as it is in other Solanaceae plants.

### Pepper accessions respond to a diversity of *P. infestans* effectors

In order to perform a primary screen of the nonhost interaction, we inoculated the infection-ready collection of 54 *P. infestans* RXLR effectors onto the leaves of 100 pepper accessions using the PVX agro-infection method (Fig. S1). The effectors are putative secreted effectors from expressed-sequence tag data of *P. infestans* and have been cloned into the binary PVX vector pGR106 for high-throughput screening of effector activities (Randall *et al*., 2005; Oh *et al*., 2009). Among 54 effectors, nine effector families including PexRD6, PexRD7, PexRD16, PexRD24, Avrblb2, PexRD41, PexRD44, PexRD49 and PexRD50 were recently determined as core effectors using criteria that measure induction of expression during infection and presence in the genome of three *P. infestans* isolates (Cooke *et al*., 2012). The pepper accessions originating from various areas including the United States, China, and Japan were selected for screening of nonhost interactions. Most of them belong to *Capsicum annuum* with a few accessions of *Capsicum chinense* (Table S2). After inoculation of RXLR effectors in duplicate, effector-induced cell death was monitored at 14 dpi. *Agrobacterium* carrying pGR106-dGFP and pGR106-PexRD2 were used as negative and positive controls, respectively. Considering the relatively low efficiency of *Agrobacterium*-mediated transient expression on pepper, we judged ‘cell death’ when either one of the duplicate inoculation sites showed cell death. Cell death confirmed twice was marked as ‘+’ (Tables 2, S2).

**Table 2:** Pepper (*Capsicum annuum*) accessions respond to a diversity of *Phytophthora infestans* effectors

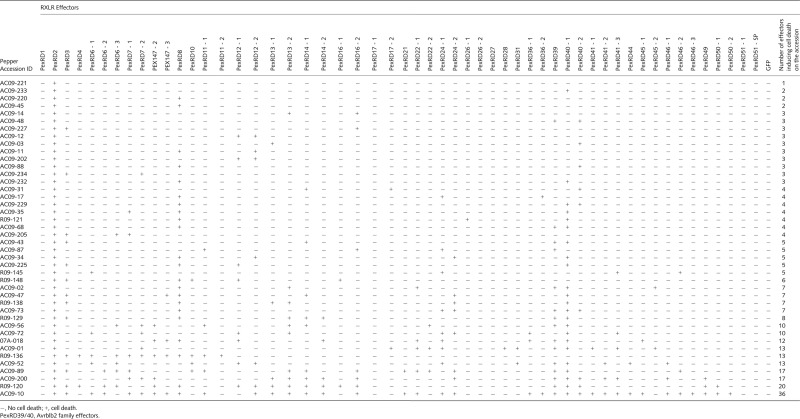

Among 100 pepper accessions, 55 showed no cell death response by inoculation of *Agrobacterium*-carrying the positive control (PexRD2), which may indicate incompatibility of these accessions with *Agrobacterium* and low growth of *Agrobacterium* resulting in insufficient expression of effectors. Three accessions (AC09-15, AC09-20 and R09-135) showed the necrotic cell-death response triggered by the negative control pGR106-dGFP, as evidenced by susceptible response to PVX developing systemic mosaic symptoms and leaf crinkling at 14 dpi (Table S2). Those three accessions were excluded for further screening. From the screening results of the other 42 accessions showing the HR against positive control PexRD2, each accession variously responded to at least one effector, showing a distributed cell death response among pepper accessions. The average number of effectors inducing cell death on each pepper accession was seven and many accessions reacted to multiple effectors. Each pepper accession showed cell death by *in planta* expression of 1–36 effectors, respectively. These results support the hypothesis that there are multiple recognitions in nonhost interactions between pepper and effectors of *P. infestans* (Table 2).

**Fig. 4:**
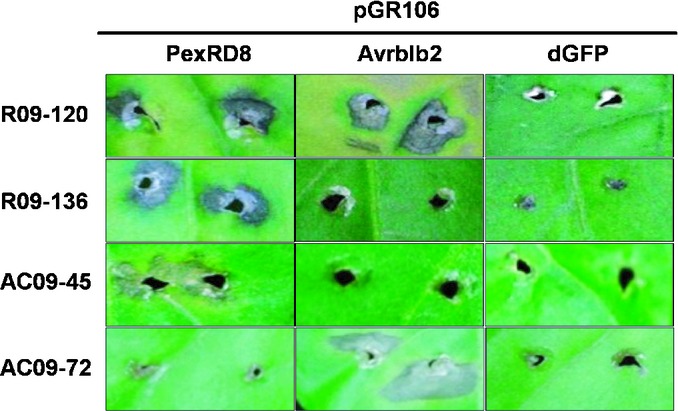
PexRD8 and Avrblb2 are recognized by a variety of pepper (*Capsicum annuum*) accessions. Hypersensitive cell death was observed in pepper accessions after wound inoculation with *Agrobacterium* carrying pGR106-PexRD8 and Avrblb2 at 14 d post-inoculation.

The RXLR effectors that interacted with pepper accessions could be divided into two groups: broadly recognized and specifically recognized effectors. The effectors recognized by a broad variety of pepper accessions included PexRD8 and PexRD39/40, known as the Avrblb2 family (Table 1; Fig. 4). Over half of the tested accessions showed the HR to those effectors. The specifically recognized effectors showed various responses, depending on the pepper accession. Generally, homologous effectors induced cell death on the same pepper accessions. For example, PexRD12-1 and PexRD12-2 exhibited 99% nucleotide sequence similarity and both triggered cell death on pepper accessions AC09-12, R09-120, AC09-202 and AC09-52. Furthermore, PexRD24-1 and PexRD24-2, sharing 94% identity, elicited cell death on pepper accessions AC09-72, AC09-73 and AC09-10. Those accessions could be postulated to have cell death-inducing factors that interact with an effector family. Taken together, these results revealed that nonhost pepper plants show cell death by interactions with multiple RXLR effectors of *P. infestans*.

### Validation of the pepper hypersensitive response to *P. infestans* effectors using recombinant PVX virions

**Fig. 5:**
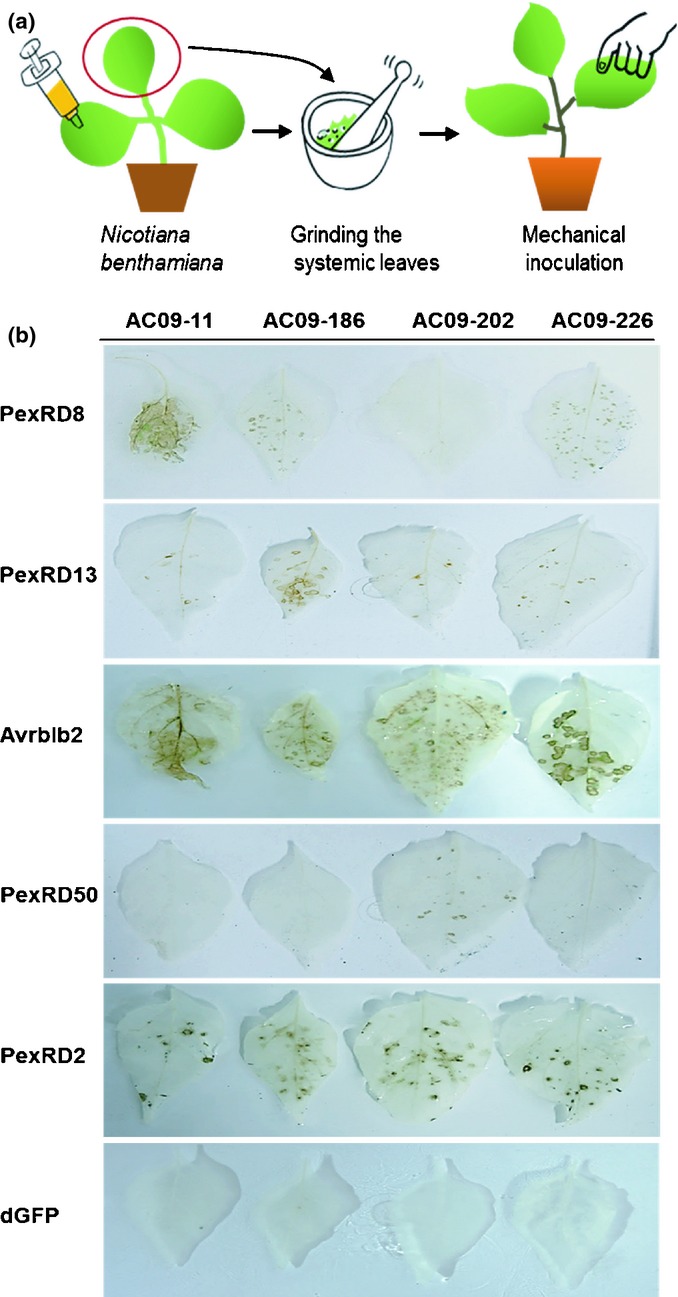
Validation of effector-induced cell death using recombinant PVX virions. (a) Overview of the PVX-mediated transient expression method is shown. *Agrobacterium* carrying RXLR effectors was inoculated into lower leaves of *Nicotiana benthamiana*. Upper leaves showing virus symptom were ground to produce a PVX-inoculum carrying RXLR effectors. The inoculum was mechanically inoculated on pepper (*Capsicum annuum*) leaves to validate the effector-induced cell death. (b) Four pepper accessions (AC09-11, 186, 202 and 226) were wound-inoculated with recombinant PVX virions (PexRD8, PexRD13, Avrblb2 and PexRD50). PVX virions of PexRD2 and dGFP were used as positive control and negative control, respectively. Inoculated leaves were harvested at 5 d post-inoculation and cleared in 100% ethyl alcohol to remove chlorophyll in order to visualize the cell death.

Some of the pepper accessions that did not show any reaction with the PVX agro-infection assay may be recalcitrant to *A. tumefaciens*-mediated transient assays. To confirm the positive interactions of pepper to *P. infestans* effectors for a further inheritance study and exclude the effect of *Agrobacterium*, we developed a PVX-virion-mediated screening method. As depicted in Fig. 5(a), recombinant PVX virions carrying RXLR effectors propagated in *N. benthami*a*na* were harvested from upper leaves that showed PVX symptoms and were manually applied on carborundum-dusted leaves of the pepper accessions. HR cell death was solely developed by PVX-mediated *in planta* expression of RXLR effectors.

Susceptible responses of pepper against PVX, including severe necrosis, have been reported in 11 cultivars of *C. annuum* species (Shi *et al*., 2008). To select accessions for the application of the recombinant virion method, we screened 14 pepper accessions for their response to PVX following inoculation of the negative control PVX-dGFP and positive control PVX-PexRD2 (Fig. S2; Table S3). Twelve out of 14 pepper accessions were randomly selected from the pepper accessions showing cell death induced by PexRD2. The other two accessions (AC09-186 and AC09-226) were chosen from the pepper accessions showing no effector-induced cell death. Upon inoculation of the recombinant virions, some accessions, including AC09-56 and AC09-200, showed cell death against PVX-dGFP, which may indicate that these accessions contain intrinsic R genes against PVX. For further study of inheritance of effector-induced cell death, we selected four pepper accessions, AC09-11, AC09-186, AC09-202 and AC09-226 showing no cell death response induced by PVX-dGFP and a clear response against PVX-PexRD2. All accessions showed cell death against PexRD2 but no cell death against PVX-dGFP (Fig. 5b).

Of 54 RXLR effectors, 13 effectors (PexRD6-3, PexRD12-1, PexRD12-2, PexRD24-2, PexRD26-1, PexRD26-2, PexRD31, PexRD39, Pex46-2, PexRD49, PexRD50-2 and PexRD51) were excluded, due to the difficulty of obtaining recombinant PVX virions. In total, recombinant PVX virions of 41 RXLR effectors were tested on four accessions (Table 3). Effector-induced cell death by PexRD8, PexRD13, Avrblb2 and PexRD50 is shown in Fig. 5(b). PexRD8 resulted in cell death on all accessions except AC09-202, confirming the results of the primary screening. Interestingly, the PexRD13-1 and Avrblb2 families (PexRD40-1 and PexRD40-2) were recognized by all four accessions. In potato, PexRD13-1 elicited cell death on *Solanum bulbocastanum* and *S. pinnatisectum* which are both resistant to *P. infestans*. Avrblb2 families induced cell death in both resistant and susceptible *Solanum* species (Vleeshouwers *et al*., 2008). These similar patterns of cell death induced by the PexRD8 and Avrblb2 families suggest that pepper has common plant factors or cognate R genes interacting with those effectors. Some effectors elicited cell death on one or two accessions. For example, PexRD24 induced cell death on AC09-11 and AC09-226. PexRD50-induced cell death was only observed on AC09-202. Overall, we confirmed the multiple interactions between pepper and effectors of *P. infestans*.

**Table 3:** Hypersensitive cell death induced by 41 RXLR effectors in pepper (*Capsicum annuum*) accessions using inoculation with recombinant PVX virions

*Phytophthora infestans*	Pepper accessions			
	
RXLR effectors	AC09-11	AC09-186	AC09-202	AC09-226
PexRD1	−	−	−	−
PexRD2	+	+	+	+
PexRD3	−	−	−	−
PexRD4	−	−	−	−
PexRD6-1	−	−	−	−
PexRD6-2	−	−	−	−
PexRD7-1	−	−	−	−
PexRD7-2	+	−	+	−
Pex147-2	−	−	−	−
Pex147-3	−	−	nd	−
PexRD8	+	+	−	+
PexRD10	+	−	−	−
PexRD11-1	−	−	−	−
PexRD11-2	−	−	−	−
PexRD13-1	+	+	+	+
PexRD13-2	+	−	+	−
PexRD14-1	−	−	−	−
PexRD14-2	−	−	−	−
PexRD16-1	−	−	−	−
PexRD16-2	+	−	+	−
PexRD17-1	−	−	−	−
PexRD17-2	−	−	−	−
PexRD21	nd	nd	+	−
PexRD22-1	−	−	−	−
PexRD22-2	−	−	−	−
PexRD24-1	+	−	−	+
PexRD27	−	−	−	−
PexRD28	−	−	−	−
PexRD36-1	+	−	−	−
PexRD36-2	−	−	−	−
PexRD40-1	+	+	+	+
PexRD40-2	+	+	+	+
PexRD41-1	+	−	−	−
PexRD41-2	nd	−	−	−
PexRD41-3	−	nd	+	−
PexRD44	−	−	−	−
PexRD45-1	−	−	−	−
PexRD45-2	−	−	−	nd
PexRD46-1	+	−	+	−
PexRD46-3	−	−	+	−
PexRD50-1	−	−	+	−

PexRD40-1/PexRD40-2, Avrblb2 family effectors.

−, No cell death; +, cell death; nd, not determined.

### Multiple loci determine responses of pepper to *P. infestans* RXLR effectors

Based on the diversity of effector-induced cell death in pepper accessions, an inheritance study was performed to determine the genetic basis of effector-induced cell death in pepper. Two accessions, AC09-226 and AC09-202, were crossed to generate an F_2_ population. Among the effectors to which these two pepper accessions reacted differentially, PexRD8 and PexRD24 caused cell death on AC09-226, whereas PexRD41-3, PexRD46 and PexRD50 induced cell death on AC09-202.

PVX virions expressing the five effectors (PexRD8, PexRD24, PexRD41, PexRD46 and PexRD50) were manually inoculated onto two leaves per plant of the F_1_ and F_2_ populations. Each F_2_ plant was treated with only one recombinant virion. Six days later, all inoculated leaves were scored for effector-induced cell death. To determine whether PVX affects the cell death induced by effectors on F_2_ plants, we applied the virion of negative control PVX-dGFP on 65 F_2_ plants, resulting in no cell death responses on any of the tested F_2_ plants. Observations of cell death induced by five effectors in F_1_ progeny indicated that plant factor(s) to determine the cell death phenotype were dominant alleles. The F_2_ distribution of PexRD8-induced cell death showed no significant deviation from the 15 : 1 ratio of cell death at the 0.05 probability level, suggesting that two unlinked dominant genes interact with the PexRD8 effector (Table 4). The F_2_ segregation of cell death induced by PexRD24, PexRD46 and PexRD50 fitted the expected 9 : 7 ratio, consistent with two independent gene loci that interact in complementary dominant epistasis to trigger effector-induced cell death. These results indicate that pepper has a set of genes that recognize a *P. infestans* RXLR effector. The cell death phenotype induced by PexRD41-3 segregated in a 3 : 1 ratio, suggesting that cell death is conditioned by a single dominant gene. Taken together, our data support the idea that multiple interactions of plant factors with effectors underpin the NHR of pepper against *P. infestans*.

**Table 4:** Multiple dominant genes mediate effector-induced cell death in F_2_ population from the cross of AC09-226 to AC09-202

		F_2_ (Pepper (*Capsicum annuum*) accessions AC09-226 × AC09-202)				
		
		Observed ratio		Expected ratio		
			
Effectors	F_1_ HR+/HR−	HR+	HR−	HR+ : HR−	Chi-square	*P*-value
PexRD8	8 : 0	141	14	15 : 1	2.047	0.152
PexRD24-1	15 : 0	101	81	9 : 7	0.042	0.837
PexRD41-3	15 : 0	101	31	3 : 1	0.162	0.687
PexRD46-1	15 : 0	83	57	9 : 7	0.524	0.469
PexRD50-1	15 : 0	27	17	9 : 7	0.468	0.494

HR, hypersensitive response-like cell death.

## Discussion

NHR is a durable resistance that includes both preformed immunity and induced immunity. Here, we report on the interaction between pepper, a Solanaceae plant, and the destructive potato late blight pathogen, the oomycete *Phytophthora infestans*. We performed a cytological investigation to examine the cellular responses of this interaction, and concluded that a localized HR is a major response of NHR in pepper against *P. infestans*. This finding is consistent with a previous study, in which NHR to *P. infestans* in *Solanum* spp. and *Arabidopsis* was associated with the HR (Vleeshouwers *et al*., 2000; Huitema *et al*., 2003). It was recently reported that PAMPs such as flagellin and the glycoprotein CBEL have elicitor activity and induce HR-like cell death (Khatib *et al*., 2004). However, the HR, a form of programmed cell death, is more frequently induced by interactions between R genes and the cognate effectors (Khatib *et al*., 2004; Naito *et al*., 2008; Coll *et al*., 2011). Based on our finding, a set of effectors of *P. infestans* were expressed during interactions with both host potato and nonhost pepper. We suggest that multiple recognition of effectors by an arsenal of R genes in pepper is an important factor in durable NHR. Pepper, tomato and potato are all in the Solanaceae family and have a relatively close evolutionary distance; thus, it seems reasonable that pepper might carry R genes that detect RXLR effectors of *P. infestans* and that ETI would therefore contribute to NHR (Schulze-Lefert & Panstruga, 2011).

As an initial step in the characterization of the interaction between RXLR effectors of *P. infestans* and pepper, we inoculated 54 PexRD effectors into 100 pepper accessions using a PVX agro-infection method. This method has been used to study the response by effectors in several *Solanum* species (Vleeshouwers *et al*., 2006, 2008) and is considered appropriate for the high-throughput screening of effector activity that induces cell death. However, pepper has a relative low efficiency of *Agrobacterium*-mediated transformation, and gene transfer by *Agrobacterium* might not be easily performed (Lee *et al*., 2004). Functional studies in pepper mainly have been conducted by gene silencing using viruses rather than overexpression. In this study, we optimized the method and confirmed the response to effectors of pepper with recombinant PVX virions for *in planta* overexpression of effectors. The method made it possible to exclude *Agrobacterium* and achieved high efficiency in a transient expression assay. For instance, pepper accessions AC09-186 and AC09-226 used for the inheritance study did not show any cell death induced by effectors using the PVX agro-infection method but clear cell death induced by effectors was observed after inoculation with recombinant PVX virions.

Several RXLR effectors of *P. infestans* are known to be recognized by R genes of *Solanum* species. R gene-avirulence effector pairs include Rpi-blb2 (Lokossou *et al*., 2009), R3a-Avr3a (Armstrong *et al*., 2005), Rpi-blb1-Avrblb1 (Vleeshouwers *et al*., 2008) and Rpi-blb2-Avrblb2 (Oh *et al*., 2009). Our finding that pepper recognized several RXLR effectors of *P. infestans*, which is a nonhost pathogen, suggests that pepper has R genes that interact with RXLR effectors from this pathogen. Based on comparative genomic analysis of *Arabidopsis* accessions, R genes were found to rapidly evolve, having higher polymorphism than plant gene families involved in basic biological processes such as ribosomal function and transcriptional regulation (Clark *et al*., 2007). Each pepper accession showed a cell death response to each RXLR effector, which could reflect a high number of polymorphisms of the R gene family among the pepper accessions. Each pepper accession exhibited cell-death responses to at least one effector, and 1–36 of the tested 54 RXLR effectors, indicating multiple interactions of the nonhost pepper with RXLR effectors. The 54 effectors used in this study account for 10% of the effectors in the *P. infestans* genome, indicating that pepper could have more interactions with effectors of *P. infestans*. As a consequence, we cannot conclude that AC09-221, showing a cell death response to only the PexRD2 effector, would be compatible to a *P. infestans* isolate not producing a PexRD2 effector. Furthermore, we identified several effectors including PexRD8 and PexRD39/40 – termed Avrblb2 – which trigger cell death on a broad range of pepper accessions. PexRD8 is known to suppress INF1-induced cell death and Avrblb2 is recognized by the late blight resistance gene *Rpi-blb2* identified from *Solanum bulbocastanum* (van der Vossen *et al*., 2005; Oh *et al*., 2009). PexRD8 and Avrblb2 also induce cell death on most of *P. infestans*-resistant wild *Solanum* species (Vleeshouwers *et al*., 2008). However, *Nicotiana benthamiana,* a member of the Solanaceae family, did not respond to either PexRD8 or Avrblb2 (Oh *et al*., 2009). These results suggest that conserved genes exist in pepper and potato that can interact with PexRD8 and Avrblb2. We also tested interactions with Avrblb2 using recombinant PVX virion in 10 pepper accessions, which included 9 out of 14 pepper accessions used in the screening of the response against PVX in addition to the reference cultivar ‘CM334’ (Table S3). Avrblb2, which is conserved in *P. infestans* isolates, induced cell death in the 10 pepper accessions tested, suggesting that pepper might have target(s) or R gene(s) with conserved functions that interact with Avrblb2. The putative R gene(s) that interact with Avrblb2 could play an important role in NHR and are likely to be distinct from Rpi-blb2 as Rpi-blb2 orthologous cloned from several pepper accessions did not recognize Avrblb2 (H.A. Lee *et al*., unpublished).

Our genetic data suggest that several major genes are associated with the interaction with *P. infestans* effectors. Those genes could be R gene(s). In a previous study, the genetic analysis of the response to *P. syringae* effectors in nonhost lettuce showed that a linkage group, containing chromosome regions including multiple R gene analogs, determines the cell death phenotype (Wroblewski *et al*., 2009). In this study, PexRD8-induced cell death in an F_2_ population from a cross between AC09-226 and AC09-202 showed a 15 : 1 phenotypic segregation ratio, indicating that two independent dominant alleles (e.g. A-B-, aaB- and A-bb) are required to confer cell death. A dual R-gene system (*RRS1-Ws* and RPS4-Ws) showed resistance to *Colleotrichum higginisianum* (Narusaka *et al*., 2009). *Arabidopsis* RPP2A and RPP2B, members of TIR-NB-LRR, are required for resistance against *Peronospora parasitica* isolate Cala2 (Sinapidou *et al*., 2004). Furthermore, the segregation of effector-induced cell death by PexRD24, PexRD41-3 and PexRD46 in the F_2_ population was consistent with a 9 : 7 ratio, suggesting that two dominant complementary genes (e.g. A-B-) are associated with effector-induced cell death. The mode of action of two putative R genes in NHR could be supported by several studies in which two R genes act cooperatively in the resistance response. The tomato *NRC1* gene encodes a coiled-coil (CC)-nucleotide binding (NB)-leucine-rich repeat (LRR) type resistance protein and involves an HR signaling pathway mediated by the Cf-4 resistance gene (Gabriels *et al*., 2007). Similarly, NRG1, encoding CC-NB-LRR, mediates resistance together with the tobacco *N* gene against *Tobacco mosaic virus* (Peart *et al*., 2005). In our experimental system, the inverse model, the interaction of an R gene with multiple effectors, could not be demonstrated. *Arabidopsis* RPM1 induces the resistance response by the interaction with two unrelated effectors of *P. syringae*, AvrB and AvrRpm1. Similarly, a dual interaction of an R gene with different effectors is likely to occur in nonhost interactions. We cannot rule out the possibility that the combination of the interactions between multiple R genes and effectors establishes durable NHR.

Our finding that pepper has the ability to respond to *P. infestans* effectors raises an interesting question. *Phytophthora infestans* is a specialized pathogen of *Solanum* spp. native to Central and South America but is known to infect neither pepper nor related *Capsicum* spp. Why, then, would the nonhost pepper carry genes that detect effectors of *P. infestans*? There are several possibilities. It is possible that pepper was formerly a host of *P. infestans* during its evolutionary history. Another explanation is that pepper does not directly respond to *P. infestans* effectors but rather to the cellular perturbations that these effectors trigger. This would be consistent with models of indirect recognition as suggested by the guard model (Innes, 2004). Recently, recognition of the *P. infestans* AVR2 effector by the *Solanum* R2 gene was shown to require an additional host protein, phosphatase BSL1 (Saunders *et al*., 2012). R2-mediated resistance to *P. infestans* was compromised specifically by the silencing of BSL1. The association of AVR2 with BSL1 and the requirement of AVR2 for interaction of R2 with BSL1 suggest the role of BSL1 as an intermediary host protein. In addition, Antonovics *et al*. (2013) hypothesized that the evolutionary mechanism for pathogen specialization by co-evolution with the host results in NHR that reduces the virulence on other potential hosts (Antonovics *et al*., 2013). These authors explained that nonhost plant species that are closely related to the host may possess the gene variants required for the resistance. However, those variants would be less exposed to selection pressure. As hypothesized, it is possible that pepper has R genes orthologous to *Solanum* species that recognize the effectors. On the basis of criteria such as sequence identity and chromosome location, 319 putative orthologous pairs of R gene families were identified from the comparative analysis between tomato and potato (Andolfo *et al*., 2013). Analysis of the pepper genome revealed that 395 NB-LRRs are clustered with those of tomato and potato. Additionally, pepper has several NB-LRRs with orthology to known Solanaceae R genes (Kim *et al*., 2014). Inversely, pepper is host to *Phytophthora capsici* which causes serious blight disease. This means that pepper R genes should have evolved to resist *P. capsici* in the evolutionary arms race. As a consequence of adaptation to *P. capsici,* pepper R genes could recognize *P. infestans* effectors that have homology with *P. capsici* effectors. Genome-wide analysis of the evolution of NB-LRRs and effectors could provide clues to elucidate the mechanism of NHR.

NHR is the most durable form of plant resistance and therefore practical and scientific interest in NHR has increased. However, it is poorly understood, partly due to interspecific genetic incompatibility. We performed functional profiling of the responses of nonhost pepper using effectors of *P. infestans* based on effector genomics. This experimental approach could possibly be exploited in other nonhost plant–pathogen interactions, which might lead to the identification of the corresponding active R genes and to the understanding of the molecular mechanism of NHR. The completion of the sequencing of the pepper genome combined with our results could offer a new approach for identifying nonhost R genes against late blight (Kim *et al*., 2014). R genes interacting with multiple effectors may confer durable resistance through functional interfamily transfer and provide a powerful source to breed broad-spectrum disease resistance.

## Supporting Information

**Fig. S1** Work-flow of this study. **Fig. S2** The response of pepper accessions against PVX upon inoculation of the negative control PVX-dGFP and positive control PVX-PexRD2.

**Table S1** The list of primers used in this study **Table S2** Cell death induced by RXLR effectors of *Phytophthora infestans* in pepper accessions **Table S3** The response of pepper accessions upon inoculation of negative control PVX-dGFP
