# Supplementary material for: Multiple recognition of RXLR effectors is associated with nonhost resistance of pepper against Phytophthora infestans
Source: New Phytol. 2014 Jun 2;203(3):926–38. doi: 10.1111/nph.12861 (PMC4143959; doi:10.1111/nph.12861)
Supplement: Supplementary file 1 [file nph0203-0926-SD1.pptm]

## Slide 1
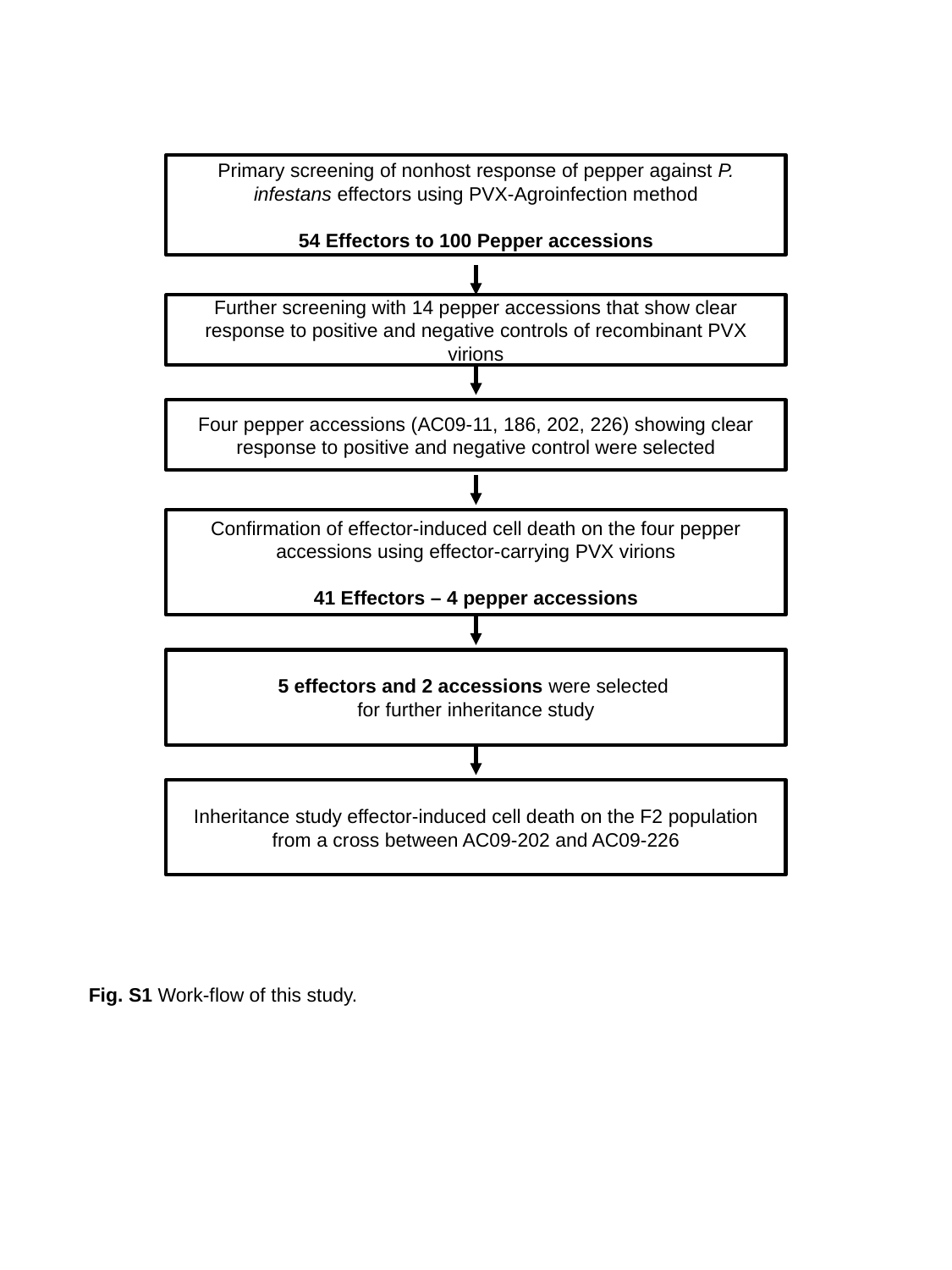

Primary screening of nonhost response of pepper against P. infestans effectors using PVX-Agroinfection method
54 Effectors to 100 Pepper accessions
Further screening with 14 pepper accessions that show clear response to positive and negative controls of recombinant PVX virions
Four pepper accessions (AC09-11, 186, 202, 226) showing clear response to positive and negative control were selected
Confirmation of effector-induced cell death on the four pepper accessions using effector-carrying PVX virions
41 Effectors – 4 pepper accessions
5 effectors and 2 accessions were selected
for further inheritance study
Inheritance study effector-induced cell death on the F2 population from a cross between AC09-202 and AC09-226
Fig. S1 Work-flow of this study.

## Slide 2
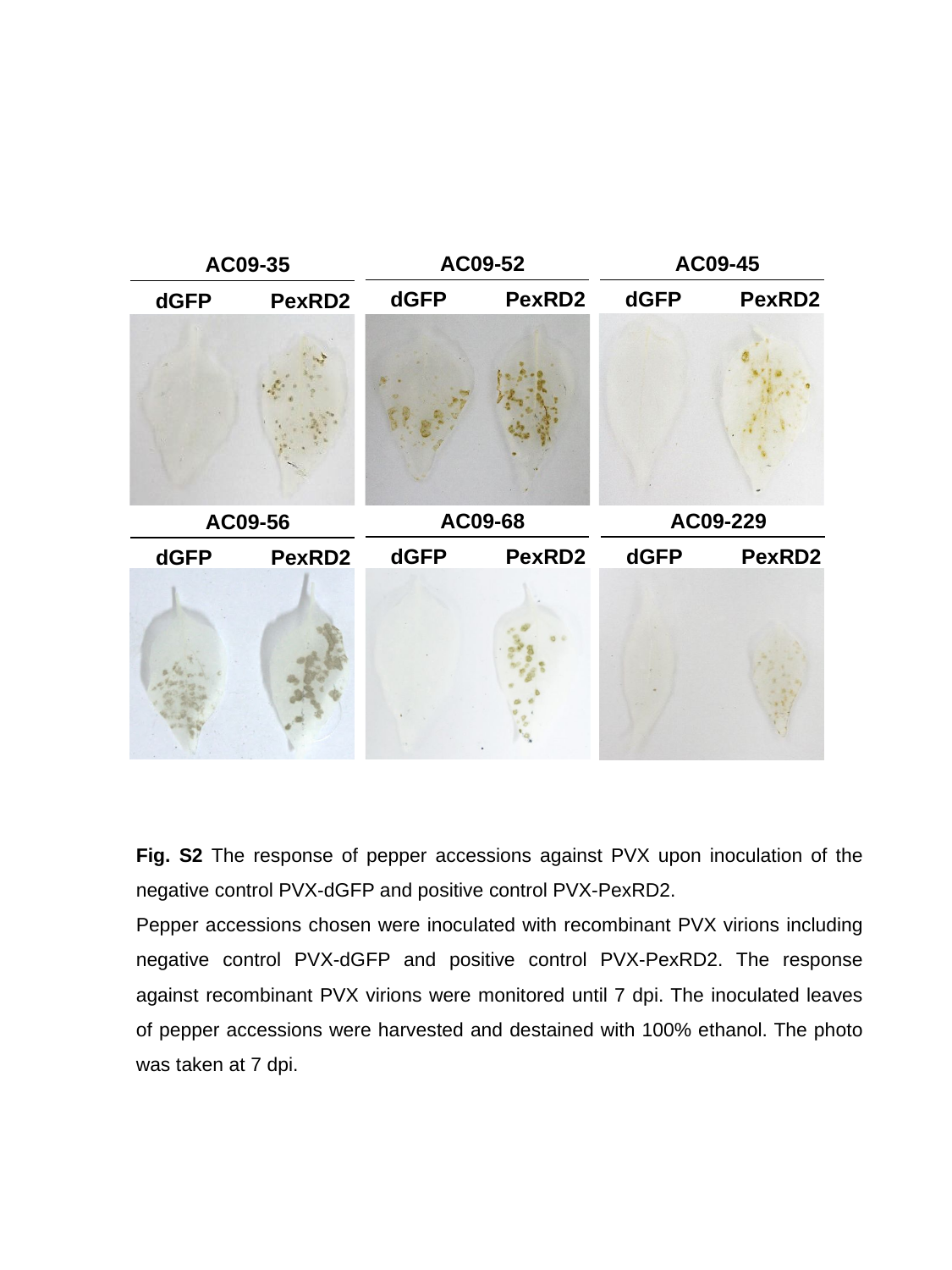

AC09-52
AC09-45
AC09-35
 dGFP PexRD2
 dGFP PexRD2
 dGFP PexRD2
AC09-68
AC09-229
AC09-56
 dGFP PexRD2
 dGFP PexRD2
 dGFP PexRD2
Fig. S2 The response of pepper accessions against PVX upon inoculation of the negative control PVX-dGFP and positive control PVX-PexRD2.
Pepper accessions chosen were inoculated with recombinant PVX virions including negative control PVX-dGFP and positive control PVX-PexRD2. The response against recombinant PVX virions were monitored until 7 dpi. The inoculated leaves of pepper accessions were harvested and destained with 100% ethanol. The photo was taken at 7 dpi.
